# Assessing the Correlation Between Disease Severity Indices and Quality of Life Measurement Tools in Pemphigus

**DOI:** 10.3389/fimmu.2019.02571

**Published:** 2019-11-06

**Authors:** Rebecca L. Krain, Carolyn J. Kushner, Meera Tarazi, Rebecca G. Gaffney, Andrea C. Yeguez, Danielle E. Zamalin, David R. Pearson, Rui Feng, Aimee S. Payne, Victoria P. Werth

**Affiliations:** ^1^Corporal Michael J. Crescenz VA Medical Center, Philadelphia, PA, United States; ^2^Department of Dermatology, University of Pennsylvania, Philadelphia, PA, United States; ^3^Department of Dermatology, University of Minnesota, Minneapolis, MN, United States; ^4^Department of Biostatistics and Epidemiology, University of Pennsylvania, Philadelphia, PA, United States

**Keywords:** pemphigus, disease severity, autoimmunity, dermatology, skin, outcome measures, autoimmune bullous disease

## Abstract

Pemphigus, an autoimmune blistering disease that affects the skin and mucous membranes, adversely impacts patients' quality of life (QOL). While there are various QOL measurement tools that can be used in this disease, few studies have assessed how a patient's change in disease severity can affect their QOL. This study aims to identify which disease severity index correlates best with the change in QOL. Fifty pemphigus patients completed QOL surveys with disease severity scored over two visits. QOL was assessed with the Autoimmune Bullous Disease Quality of Life (ABQOL), Dermatology Life Quality Index (DLQI), Skindex-29, and Short Form Survey 36 (SF-36). Disease severity was scored with the Pemphigus Disease Area Index (PDAI) and Autoimmune Bullous Skin Disorder Intensity Score (ABSIS). Correlations between the change in QOL scores and change in disease severity were analyzed using Spearman's coefficient (*r*). The change in PDAI showed a strong correlation (*r* = 0.60–0.79) with changes in the ABQOL, Skindex-29 symptoms (Skindex-S), and Skindex-29 functioning (Skindex-F) subscales for all patients (*n* = 50). For patients with mucosal disease (*n* = 24), the change in PDAI showed a strong correlation with changes in the ABQOL and Skindex-S subscale. For patients without mucosal disease, the change in PDAI showed a strong correlation with the Skindex-S. The change in ABSIS showed a strong correlation with Skindex-S for all patients and patients with no mucosal involvement, but showed no strong correlations for patients with mucosal involvement. The changes in PDAI always had a stronger correlation than the changes in ABSIS scores to changes in the ABQOL, DLQI, and Skindex-29 subscales, except where the PDAI and ABSIS scores were about the same for the Skindex-S subscale in patients with no mucosal involvement (*r* = 0.76 and *r* = 0.77, respectively). PDAI is superior to ABSIS in its correlation with validated QOL tools. The QOL tools that appear to be of most use in clinical trials and patient management are the Skindex-S and ABQOL.

## Introduction

Pemphigus is a rare but serious autoimmune blistering disease caused by autoantibodies against desmosomes (1, 2). Although a previously deadly diagnosis because of skin barrier breakdown leading to infection, the advent of steroids and steroid-sparing agents have allowed pemphigus to be considered a less-fatal, chronic disease (3). Therefore, assessing a pemphigus patient's quality of life (QOL) has become an important part of monitoring the disease (3). Despite the limited literature on QOL in pemphigus (3–12), it is clear that this disease greatly affects patients' emotions, physical health, and social functioning (12, 13). The number of clinical trials in pemphigus have increased immensely over the past decade (14), creating a growing need for objective measurements in both QOL and disease severity to monitor improvement.

Health related QOL outcomes are becoming important measurements when conducting clinical trials and assessing new pharmaceutical treatments (15). Therefore, multiple QOL measurement tools across all fields, including dermatology, have been created. QOL outcome measurements can also be useful for physicians, patients, and health insurers when deciding on therapeutic options and allowing for shared-decision making (15). In regard to QOL in pemphigus, dermatologists have used general health QOL measurement tools including the Short Form Survey 36 (SF-36), as well as dermatology specific tools such as the Dermatology Life Quality Index (DLQI) and the Skindex-29 (12, 16). An autoimmune blistering disease-specific QOL measurement tool has also been created, the Autoimmune Bullous Disease Quality of Life (ABQOL) (3, 6).

In addition to the various QOL assessment tools, there are also two commonly used validated objective disease severity indices: the Pemphigus Disease Area Index (PDAI) and the Autoimmune Bullous Skin Disorder Intensity Score (ABSIS) (17–19). Just as it is important to have a validated QOL measurement tool for clinical trials, there is a similar need for understanding the properties of QOL measures in blistering diseases and to have an established disease severity index to compare and assess results across the field.

The purpose of this study is to evaluate which disease severity score correlates best with the change in QOL scores. This may help in clinical trial design when choosing a measurement tool that captures both disease severity and QOL.

## Patients and Methods

Patients with the appropriate clinical presentation, histological findings on biopsy, and at least one positive immunochemical test consistent with pemphigus (direct or indirect immunofluorescence, and/or ELISA), provided written consent to study participation in a prospective autoimmune blistering disease database at the Hospital of the University of Pennsylvania (HUP) between April of 2016 and February of 2019. The study protocol was approved by the institutional review board. Of the 107 patients in our database who were diagnosed with pemphigus vulgaris (PV) or pemphigus foliaceus (PF), 50 patients completed both an enrollment and a follow-up visit. Patients with no disease activity at both enrollment and follow-up visits were not included in the study. Subcategorization was performed for patients with mucosal disease and patients without mucosal disease. Patients with mucosal disease had documented mucosal findings at enrollment and/or follow-up visits. Patients without mucosal disease did not have mucosal findings at both enrollment and follow-up visits, regardless of a prior history of mucosal involvement. At each visit, patients completed four QOL forms: the ABQOL, DLQI, Skindex-29, and SF-36. Patients' skin was examined and assessed during the visits using the PDAI and the ABSIS scoring systems. Patient demographics, including age, sex, and disease type, were recorded.

### Pemphigus Disease Area Index (PDAI)

The PDAI scores disease activity based on anatomical location and the size of lesions. A maximum of 120 points are available to score skin activity excluding the scalp, 10 for scalp activity, and 120 points for mucosal activity, with a maximum score of 250 points (20–22). Damage or post-inflammatory hyperpigmentation is scored separately from the activity score, with a maximum of 13 points available (20–22). The PDAI has been validated and shown to have an intra-rater intra-class correlation coefficient (ICC) of 0.98 and an inter-rater ICC of 0.86 for skin activity (23–25).

### Autoimmune Bullous Skin Disorder Intensity Score (ABSIS)

The ABSIS ranges from 0 (no activity or damage) to 206, with 150 points for skin activity or damage, 11 for mucosal involvement, and 45 for oral pain or bleeding (a subjective/patient reported measurement). The ABSIS score combines disease activity and damage together, by scoring the quality of the skin lesion (26). The ABSIS applies the “rule of nines,” which measures amount of disease by body surface area (BSA), where the palm of the patient's hand is one BSA (26). The percent BSA is multiplied by whether the lesion is re-epithelialized (0.5), dry and erosive (1.0), or exudative and erosive (1.5) (21, 22, 26). The lowest BSA score that can be assigned is a 1, even if a patient only has one small lesion with a BSA of 0.1%. Therefore, using this example, if this patient had an exudative lesion with a BSA of 0.1%, then the score would be 1.5 (1% BSA × 1.5), rather than 0.15 (0.1% BSA × 1.5) (23).

### Quality of Life Measurement Tools

Many QOL measurement tools have been developed to assess various diseases. The SF-36 is a form used for all disease entities, not limited to dermatology. It looks at eight different QOL categories: physical functioning, role limitations due to physical health, role limitations due to emotional problems, energy/fatigue, emotional well-being, social functioning, pain, and general health. Higher SF-36 scores indicate a more favorable QOL (27).

The DLQI and the Skindex-29 are both QOL measurement tools developed specifically for dermatologic diseases. The DLQI is a 10-question survey: scores range from 0 to 30, with a higher score indicating worse QOL. Questions concern topics such as patients' symptoms and feelings, daily activities, leisure, personal relationships, work and school, as well as treatment (12, 28). The Skindex-29 has 29 questions split into three subscales: Skindex-29 symptoms (Skindex-S), emotions (Skindex-E), and functioning (Skindex-F) (29, 30).

The ABQOL is a 17 question QOL measurement tool specific for autoimmune blistering diseases that was developed to capture issues related to the disease that may not be reflected in skin-specific instruments (31). The ABQOL has shown reliable internal constancy and test-retest reliability, with an intra-class correlation coefficient of 0.93 (95% confidence interval, 0.88–0.94) (3).

### Statistical Analysis

The difference, or change, in score for each QOL measurement tool, as well as the difference in disease severity index scores, were calculated from enrollment to follow-up visits. The correlation between change in QOL scores and change in disease severity scores were analyzed using Spearman's correlation coefficient (r) for all combinations of the QOL measurement tools and the disease severity indices (PDAI and ABSIS). Spearman correlation coefficient cutoffs for very weak, weak, moderate, strong, and very strong were the absolute value of 0.0–0.19, 0.20–0.39, 0.40–0.59, 0.60–0.79, and 0.80–1.0 respectively, which has been classified by the *British Medical Journal* guidelines (32). This analysis was completed for all patients, as well as for the subcategorization of patients who had mucosal involvement at some point during their two visits and patients who did not have mucosal involvement during their visits. GraphPad Prism was used to conduct the analyses.

## Results

**Figure 1:**
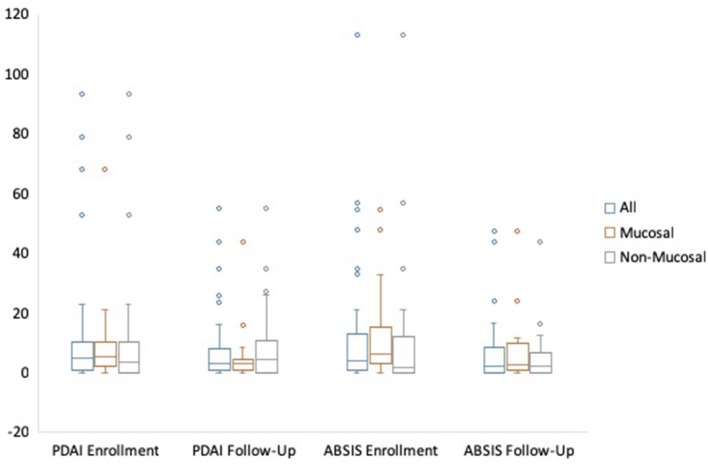
Median PDAI and ABSIS with IQR from enrollment to follow-up visit.

A total of 50 patients, 43 (86%) with PV and 7 (14%) with PF completed both an enrollment and follow-up visit. Twenty-four (48%) patients had mucosal involvement at some point during their two visits, and 26 (52%) did not have mucosal involvement. Further patient characteristics are summarized in Table 1. Median time from disease onset to enrollment visit was 2.8 years (IQR = 0.8–5.4) for all patients, 1.9 years for patients with mucosal involvement, and 4.5 years for patients with no mucosal involvement. Median time from enrollment to follow-up visit was 0.45 years. Median PDAI scores at enrollment for all patients, patients with mucosal disease, and those without mucosal disease were 5.00, 5.30, and 3.50 respectively, while median PDAI scores during follow-up were 3.00 for all patients and patients with mucosal disease, and 4.30 for patients without mucosal disease. Median ABSIS scores at enrollment for all patients, patients with mucosal disease, and those without mucosal disease were 4.00, 6.28, and 1.50, while median ABSIS scores during follow-up were 2.00, 2.50, and 2.00, respectively (Figure 1 and Table 2).

**Table 1:** Patient characteristics.

	**All (*n* = 50)**
**Age**	
<45	8 (16%)
45-64.9	22 (44%)
≥65	20 (40%)
**Sex**	
Male	24 (48%)
Female	26 (52%)
**Diagnosis**	
Pemphigus Foliaceus	7 (14%)
Pemphigus Vulgaris	43 (86%)
**Mucosal Involvement**	
Yes	24 (48%)
No	26 (52%)
**Race**	
White	37 (74%)
African American or Black	3 (6%)
Asian	8 (16%)
Other	2 (4%)

**Table 2:** Median PDAI score and IQR.

	**All**		**Mucosal**		**Non-mucosal**	
	**Median score**	**IQR**	**Median score**	**IQR**	**Median score**	**IQR**
PDAI Enrollment	5.00	1.25–10	5.30	2.98–10.15	3.50	0.00–9.33
PDAI Follow-up	3.00	1.00–8.00	3.00	1.00–4.08	4.30	0.50–9.58
ABSIS Enrollment	4.00	1.00–12.38	6.28	3.00–14.00	1.50	0.00–12.00
ABSIS Follow-up	2.00	0.10–7.50	2.50	1.00–9.56	2.00	0.00–5.75

*PDAI, Pemphigus Disease Area Index; ABSIS, Autoimmune Bullous Skin Disorder Intensity Score; IQR, interquartile range*.

For all patients (*n* = 50), the change in PDAI showed a strong correlation (*r* = 0.60–0.79) with changes in the ABQOL (*r* = 0.60), Skindex-S (*r* = 0.75), and Skindex-F (*r* = 0.61) subscales. For patients with mucosal disease (*n* = 24), the change in PDAI showed a strong correlation with changes in the ABQOL (*r* = 0.67) and Skindex-S subscale (*r* = 0.62). For patients without mucosal disease during the two visits, the change in PDAI showed a strong correlation with the Skindex-S subscale (*r* = 0.76). The change in ABSIS displayed a strong correlation with Skindex-S for all patients (*r* = 0.73) and patients with no mucosal involvement (*r* = 0.77), but showed no strong correlations for patients with mucosal involvement (Table 3).

**Table 3:** Correlation using Spearman's correlation coefficient (*r*) of the change in quality of life score with the change in disease severity from enrollment to follow-up visit.

	**PDAI**			**ABSIS**		
	**All (*n* = 50)**	**Mucosal Involvement (*n* = 24)**	**No Mucosal Involvement (*n* = 26)**	**All (*n* = 50)**	**Mucosal Involvement (*n* = 24)**	**No Mucosal Involvement (*n* = 26)**
ABQOL	0.60	0.67	0.56	0.51	0.38	0.49
DLQI	0.58	0.50	0.58	0.46	0.29	0.51
**Skindex-29**						
Symptoms	0.75	0.62	0.76	0.73	0.52	0.77
Emotions	0.45	0.29	0.59	0.37	0.17	0.51
Functioning	0.61	0.40	0.56	0.49	0.28	0.44
**SF-36**						
General health	−0.14	−0.11	−0.19	−0.11	−0.0053	−0.25
Physical functioning	−0.23	−0.17	−0.25	−0.16	−0.0043	−0.33
Role limitations (physical health)	−0.38	−0.29	−0.37	−0.37	−0.15	−0.37
Role limitations (emotional problems)	−0.19	0.0093	−0.29	−0.27	−0.056	−0.38
Energy/fatigue	−0.41	−0.59	−0.24	−0.40	−0.44	−0.27
Emotional well-being	−0.33	−0.31	−0.23	−0.21	0.039	−0.24
Social functioning	−0.27	−0.10	−0.22	−0.28	−0.19	−0.17
Pain	−0.33	0.079	−0.58	−0.45	−0.16	−0.63

*PDAI, Pemphigus Disease Area Index; ABSIS, Autoimmune Bullous Skin Disorder Intensity Score; ABQOL, Autoimmune Bullous Disease Quality of Life; DLQI, Dermatology Life Quality Index; SF-36, Short Form Survey 36*.

The eight different SF-36 subscales as well as the DLQI primarily displayed weak to moderate correlations to both the PDAI and ABSIS. For all patients (*n* = 50), the change in the SF-36 role limitations due to physical heath, energy/fatigue, and pain subscales showed weak to moderate correlations with the change in PDAI (*r* = −0.38, −0.41, and −0.33, respectively) and ABSIS (*r* = −0.37, −0.40, and −0.45, respectively). A weak correlation was also found with the change in the SF-36 emotional well-being subscale to the change in PDAI (*r* = −0.33). For patients with mucosal disease (*n* = 24), the change in the SF-36 energy/fatigue subscale showed moderate correlations with the change in PDAI (*r* = −0.59) and ABSIS (*r* = −0.44). For patients with no mucosal disease (*n* = 26), the change in SF-36 pain subscale showed a moderate correlation with the change in PDAI (*r* = −0.58), and a strong correlation with the change in ABSIS (*r* = −0.63) (Table 3). In regard to the change in PDAI, the DLQI showed moderate correlations to all patients, patients with mucosal involvement, and no mucosal involvement (*r* = 0.58, 0.50, and 0.58, respectively). Similarly, in regard to the ABSIS, all patients and patients with no mucosal involvement remained in the moderate range when correlating to the DLQI, however, patients with mucosal involvement had a weak correlation of the ABSIS to the DLQI (*r* = 0.29).

The Skindex-29, specifically the Skindex-S, and ABQOL displayed generally stronger correlations than the other measurement tools. Strong correlations were found with the change in Skindex-S to both the PDAI and ABSIS for all patients (*r* = 0.75 and *r* = 0.73, respectively), patients with no mucosal involvement (*r* = 0.76 and 0.77, respectively), and patients with mucosal involvement for PDAI only (*r* = 0.62) (Table 3). A moderate correlation was noted for the Skindex-S and ABSIS for patients with mucosal involvement (*r* = 0.52). The Skindex-E and Skindex-F showed weak and moderate correlations to the PDAI and ABSIS, except for a strong correlation with the change in Skindex-F and change in PDAI for all patients (*r* = 0.61) (Table 3). For the ABQOL, strong correlations were found with the change in PDAI for all patients (*r* = 0.60) and patients with mucosal involvement (*r* = 0.67). The change in ABQOL also showed moderate correlations with the change in ABSIS for all patients (*r* = 0.51) and patients with no mucosal involvement (*r* = 0.49), and with the change in PDAI in patients with no mucosal involvement (*r* = 0.56). A weak correlation was noted for patients with mucosal involvement in regard to the ABSIS (*r* = 0.38). There were no strong correlations found with the DLQI; however, moderate correlations were noted for each category, except for a weak correlation with the ABSIS for patients with mucosal involvement (*r* = 0.29) (Table 3).

## Discussion

Treatment options for pemphigus have greatly improved over the past two decades (13). Nonetheless, there is a high unmet need for FDA-approved therapies, and many agents have entered, or are entering, clinical trials for pemphigus. To optimize these trials, standard measurement tools should be used to assess skin disease severity as well as QOL. Assuming QOL decreases with skin severity, we conducted this study to identify which disease severity index correlates best with QOL.

Our data revealed that the SF-36 did not correlate strongly with the PDAI or ABSIS for the majority of analyses (Table 3). Most SF-36 and DLQI correlations were weak or moderate, except for the SF-36 pain subscale. We hypothesize that the SF-36 showed generally weak correlations because it addresses overall health rather than specific dermatologic symptoms. This relates to our patient population, where 40% are over the age of 65 and likely suffering from other health conditions or limitations of older age (33, 34). The age group of our cohort (median age = 60) is consistent with the general pemphigus population, who tend to present in their fifth to sixth decade of life (35).

Assessment of the QOL measurement tools showed largely strong correlations with the Skindex-S subscale to both the PDAI and ABSIS for all patients as well as patients with and without mucosal disease, demonstrating its use, and potential preference, in patient management and clinical trials (Table 3). On the contrary, the Skindex-S unexpectedly showed only a moderate, rather than strong, correlation to the ABSIS for patients with mucosal disease (*r* = 0.52). This finding was surprising, as the ABSIS contains QOL questions regarding mucosal involvement (pain and bleeding). It was therefore believed that a score that addressed oral symptoms would show a strong correlation with the Skindex-S subscale score for patients with mucosal disease. However, of the 206 possible points in the ABSIS, only 56 of those points directly address mucosal involvement (11 points) as well as subjective oral pain and bleeding (45 points). In contrast, the PDAI allocates more points toward mucosal involvement (120 out of 250 total points). Therefore, although the ABSIS may include QOL questions, this aspect is only a minor part of the disease severity index.

The ABQOL was also found to show strong correlations with the PDAI in regard to all patients (*r* = 0.60) and patients with mucosal involvement (*r* = 0.67), and was the strongest correlation found in patients with mucosal disease. It is likely that the ABQOL correlated best with the PDAI for patients with mucosal disease because this disease-specific QOL measurement tool directly addresses painful erosions in the mouth. For instance, 3 of the 17 questions in the ABQOL assess for mucosal involvement, compared to the other QOL measurement tools, like the DLQI, that only emphasize skin disease, which patients often interpret as not including the mucosa. The ABQOL assessment may therefore be a useful tool in clinical trials for pemphigus patients with mucosal involvement, such as PV specific trials.

Correlations of the change in PDAI were superior to those of the ABSIS in regard to the change in the majority of QOL tools. Looking at all patients, those with mucosal involvement, and those without, there were a total of 13 analyses conducted for the ABQOL, DLQI, the three subscales of the Skindex-29, and the eight subscales of the SF-36 in correlation with the PDAI and ABSIS. Ten of the 13 (76.9%) analyses showed a stronger correlation with the PDAI over the ABSIS. The analysis that showed a stronger correlation with the ABSIS was the Skindex-S for patients without mucosal involvement, although the ABSIS and PDAI values were about the same (*r* = 0.77 and *r* = 0.76, respectively). While some correlations did not vary greatly between the two severity tools, the fact that the PDAI had largely stronger correlations suggests that the PDAI may be better at capturing disease activity that correlates with QOL. This finding is consistent with a prior study that showed ABSIS data to be skewed to the left (lower scores) as compared to the PDAI (23). The lower scores seen in the ABSIS support its limitation in its ability to evaluate changes in skin disease severity in patients with mild disease and, given the generally mild disease in our cohort, may be why this index did not correlate well with the changes in QOL measurement tools.

This study had several strengths as well as limitations. The strengths included data collection in a prospective manner and a relatively large pemphigus database, although the cohort size was small for the statistical analyses. Additionally, this study included a patient population similar to those who may be eligible to enter future pemphigus clinical trials. The limitations included a retrospective analysis, a cohort from a single institution that is a tertiary center, and the absence of data on comorbid conditions when assessing the QOL tools.

Our cohort had relatively mild disease, with low PDAI and ABSIS scores, which may have prevented our results from being fully applicable to patients with moderate to severe disease. Patients typically present with highly active disease for a short period of time because steroids are fast-acting and effective, lowering disease activity by their first follow-up visit. However, our first study visit was not always with new patients, and many may have been well-controlled by the time they were enrolled in our database. At initial visit for our database, 14 patients displayed a PDAI of ≥9 and 7 patients had a PDAI of ≥15, which are considered two different “moderate” disease cutoff points (17, 36). Only 4 patients displayed both a PDAI of ≥25 and ≥45, two different “severe” disease cutoff points (17, 36). Our results may therefore have been different if we gathered data at the peak of their disease. Often, however, patients who are enrolled in clinical trials have been initially controlled on steroids or other first-line treatments prior to receiving further therapy or interventions.

Our study identified disease severity index and QOL measurement tools that should be used in clinical trials and patient management. The PDAI and the Skindex-S subscale of the Skindex-29 showed excellent promise for use in such settings, in a patient population that is representative of those seen during routine practice at a tertiary referral medical center. Future studies of the ABQOL in patients with mucosal involvement and in those with higher disease activity scores may further elucidate the utility of the various QOL measurement tools in relation to disease activity.

## Data Availability Statement

The datasets for this manuscript are not publicly available because this is a prospective database of patients with autoimmune blistering diseases who consented to participate in the database and who remain unidentifiable. Requests to access the datasets should be directed to VW, werth@pennmedicine.upenn.edu.

## Author Contributions

RK, CK, and VW contributed to the conception and design of the study and wrote the manuscript. RK, CK, AY, and DZ organized the database. RK, CK, RG, MT, RF, DP, AP, and VW analyzed and interpreted the dataset. All authors contributed to manuscript revision, read, and approved the submitted version.

### Conflict of Interest

AP is a co-founder and equity holder in Cabaletta Bio, Inc., focused on targeted immunotherapy of pemphigus. She is an inventor on patents licensed by Novartis and Cabaletta Bio for cellular immunotherapy of autoimmune diseases, and has previously served as a consultant for Syntimmune, Inc. VW has grants from Roche/Genentech and Syntimmune. She is a consultant for Roche/Genentech, Syntimmune, Janssen, and Principia. VW was involved in the development of the PDAI. The remaining authors declare that the research was conducted in the absence of any commercial or financial relationships that could be construed as a potential conflict of interest.
